# A Comparative Study of Pulmonary Function Tests in Recreational Swimmers, Recreational Runners, and Sedentary Healthy Male Adults

**DOI:** 10.7759/cureus.102382

**Published:** 2026-01-27

**Authors:** Tauseef T Husain, Manoj T Jiwtode, Ranjana C Shingne

**Affiliations:** 1 Department of Physiology, Government Medical College, Buldhana, IND; 2 Department of Physiology, Indira Gandhi Government Medical College, Nagpur, IND

**Keywords:** aerobic exercise, pulmonary function tests, running, spirometry, swimming, young adults

## Abstract

Background: Regular aerobic exercise is associated with variations in pulmonary function through adaptations in respiratory muscles, lung volumes, and ventilatory efficiency. Swimming and running are commonly practiced aerobic activities that show differences in their physiological and mechanical characteristics related to respiration. However, limited data are available comparing pulmonary function among recreational swimmers, recreational runners, and sedentary individuals within a narrow age group.

Objectives: To compare pulmonary function tests among recreational swimmers, recreational runners, and sedentary healthy male adults aged 18-25 years.

Methodology: This cross-sectional comparative study included healthy male participants aged 18-25 years, divided into three groups: recreational swimmers, recreational runners, and sedentary controls. Participants in the swimming and running groups reported engaging in their respective activities for approximately one hour per session, four to five times per week. Detailed assessment of training intensity, swimming stroke type, running pace, and environmental conditions was not performed. Pulmonary function tests, including forced vital capacity (FVC), forced expiratory volume in one second (FEV₁), peak expiratory flow rate (PEFR), and maximal voluntary ventilation (MVV), were measured using standard spirometric techniques. Data were analyzed using appropriate statistical tests, and a *P*-value <0.05 was considered statistically significant. The study design focused on activity-based grouping rather than detailed sport-specific training characteristics.

Results: Pulmonary function parameters were significantly higher in recreational swimmers and recreational runners compared to sedentary controls. Recreational swimmers demonstrated the highest values of FVC, FEV₁, PEFR, and MVV, followed by recreational runners, while sedentary participants exhibited the lowest values. The differences observed among the three groups were statistically significant.

Conclusions: Regular participation in recreational swimming and running shows a positive association with improved pulmonary function compared to a sedentary lifestyle in healthy young adult males. Recreational swimming demonstrates a stronger relationship with higher pulmonary function values than running, possibly related to the unique respiratory demands associated with water immersion and controlled breathing patterns.

## Introduction

Pulmonary function tests (PFTs) are widely used to assess respiratory health, ventilatory efficiency, and functional lung capacity in both clinical and research settings. Lung volumes and airflow parameters are influenced by multiple factors, including age, sex, body composition, and habitual physical activity. While regular aerobic exercise is associated with favorable respiratory adaptations, the magnitude and nature of these changes may vary according to the type, intensity, and training status of physical activity.

Physically active individuals often demonstrate superior pulmonary function compared with sedentary populations. However, important distinctions exist between elite athletes, recreational exercisers, and sedentary individuals, and findings from elite athletic populations may not be directly generalizable to recreationally active or general populations. In the present context, recreational training refers to regular, non-professional physical activity performed for health and fitness purposes, without participation in elite or competitive athletic programs or structured high-performance training. Vedala et al. reported significantly higher forced vital capacity (FVC) and forced expiratory volume in one second (FEV₁) among physically active individuals, emphasizing the positive association between regular physical activity and respiratory efficiency [1]. These adaptations are thought to reflect improvements in respiratory muscle performance, thoracic compliance, and ventilatory mechanics.

Swimming has been extensively studied for its unique respiratory adaptations. Controlled breathing patterns, intermittent breath-holding, hydrostatic pressure, and the horizontal body position during swimming promote greater lung expansion and respiratory muscle conditioning. Akhade and Muniyappanavar observed significantly higher lung volumes in swimmers compared with sedentary controls, suggesting sport-specific pulmonary adaptations [2]. However, much of the existing literature focuses on trained or competitive swimmers, and less is known about these adaptations in recreational swimmers.

Running, as a land-based aerobic activity, places sustained demands on the respiratory system. Regular running increases minute ventilation, strengthens respiratory muscles, and improves ventilatory efficiency through repeated rhythmic breathing at higher metabolic loads. Studies involving moderate aerobic exercise have shown that individuals engaged in activities such as running exhibit improved spirometric parameters compared with sedentary individuals. Okon et al. reported favorable pulmonary function indices among young adults participating in regular moderate aerobic exercise, supporting the beneficial role of recreational land-based endurance activities [3].

In addition to lung volumes, respiratory muscle strength is an important determinant of pulmonary performance. Segizbaeva and Aleksandrova demonstrated that trained individuals have significantly greater inspiratory and expiratory muscle strength compared with untrained persons, contributing to enhanced ventilatory outcomes during physical activity [4]. Comparative studies between athletes and non-athletes further confirm superior pulmonary function among those engaged in regular training; however, these findings often reflect elite or highly trained cohorts and may not fully represent recreational exercise populations [5].

Differences between aquatic and land-based training modalities have also been documented. Doherty and Dimitriou demonstrated that swimmers exhibit greater lung volumes than land-based athletes and sedentary individuals using allometric scaling, highlighting the distinct ventilatory demands of swimming compared with running and sedentary behavior [6].

However, there is a paucity of studies that simultaneously compare recreational swimmers and recreational runners within a uniform young adult age group, using standardized spirometric outcome measures and a consistent cross-sectional methodology, while explicitly excluding elite or competitive athletes.

Despite existing evidence, comparative data specifically evaluating recreational swimmers, recreational runners, and sedentary healthy male adults remain limited. This represents an important knowledge gap with clinical relevance for the interpretation of PFTs in physically active but non-elite populations. Therefore, the present study aims to compare pulmonary function parameters among recreational swimmers, recreational runners, and sedentary healthy male adults to better characterize exercise-specific respiratory adaptations in a recreational setting.

## Materials and methods

Study design and timeline

This was a cross-sectional observational study conducted in the Department of Physiology at a tertiary care medical institution. The study was conducted during the period from September 2018 to August 2020.

Study population

A total of 150 healthy male volunteers aged 18-25 years were recruited for the study. Participants were divided into three groups based on habitual physical activity patterns.

For the purpose of this study, recreational training was defined as regular, non-professional physical activity performed for health and fitness purposes, without participation in elite or competitive athletic programs and without structured high-performance training.

Swimmers (n = 50)

Participants who had been regularly practicing swimming for the past two years, with a training frequency of five sessions per week, each lasting 1-2 hours, and who were not involved in professional or competitive swimming.

Runners (n = 50)

Individuals performing regular running exercise with a similar frequency and duration, not engaged in professional athletic training or competitive running.

Sedentary controls (n = 50)

Individuals not involved in any regular structured physical activity.

All participants were recruited from the same urban geographic location to ensure uniform environmental exposure.

Inclusion and exclusion criteria and health verification

Participant health status was verified through detailed medical history and physical examination conducted by a qualified physician. This included assessment for respiratory symptoms, past medical history of cardiopulmonary disease, smoking history, recent respiratory infections, and general systemic illness. Only individuals deemed clinically healthy on history and examination were included.

Pulmonary Function Testing (Instrumentation and Procedures)

Pulmonary function testing was performed under standardized laboratory conditions using standard spirometric procedures as described in previous studies [5]. Spirometry was recorded using an RMS Helios 401 spirometer (transducer no. 400-666) with disposable mouthpieces and a nose clip. The spirometer met American Thoracic Society (ATS) criteria and was volume calibrated daily [7]. All gas volumes were automatically corrected to Body Temperature and Pressure Saturated (BTPS) by the instrument.

Participants were familiarized with the procedure and allowed adequate rest before testing. All tests were conducted in the sitting position, with participants seated upright and wearing a nose clip to prevent nasal air leakage.

A single reusable nose clip was used for all participants and was cleaned and disinfected with 70% isopropyl alcohol before and after each use. Disposable mouthpieces were used for each participant.

The pulmonary parameters measured included forced vital capacity (FVC), forced expiratory volume in one second (FEV₁), FEV₁/FVC ratio, maximum voluntary ventilation (MVV), and peak expiratory flow rate (PEFR). Each participant performed three acceptable and reproducible maneuvers, and the highest value was selected for analysis.

Sample size calculation

Sample size was determined based on expected differences in maximum voluntary ventilation (MVV) between physically active and sedentary individuals, using standard sample size estimation methods with the following assumptions: mean difference (μd) = 10.52, power (1 − *β*) = 80%, *α* error = 5%, standard deviation in swimmers (*S*_1_) = 17.49, and standard deviation in controls (*S*_2_) = 15.47. Pooled variance was calculated as:

\[ S_p^{2} = \frac{S_1^{2} + S_2^{2}}{2} \]

Pooled variance was used only for sample size estimation and not as an analytical effect size measure.

Sample size was calculated using the formula:

\[ n = \frac{2 S_p^{2}\,(Z_{1-\alpha/2} + Z_{1-\beta})^{2}}{\mu_d^{2}} \]

The required sample size per group was *n* = 50, as obtained using n-Master software version 2.0 (Christian Medical College (CMC), Vellore, India). Accordingly, 150 male subjects were included in the study (50 swimmers, 50 runners, and 50 sedentary healthy adults).

Anthropometric measurements

Anthropometric parameters, including age, height, weight, body mass index (BMI), waist circumference, hip circumference, and waist-to-hip ratio, were measured using standardized protocols. BMI was calculated as weight in kilograms divided by height in meters squared. These parameters were compared across the three groups to ensure baseline comparability (Table 1).

**Table 1:** Age and anthropometric characteristics distribution among various groups. Values are expressed as mean ± standard deviation (SD). Intergroup comparison was performed using one-way analysis of variance (ANOVA). NS indicates not statistically significant. *Statistically significant differences (*P* < 0.05). BMI, body mass index

Variables	Group I (Swimmers) (Mean ± SD)	Group II (Runners) (Mean ± SD	Group III (Sedentary) (Mean ± SD)	*F*-value	*P*-value*
Age (years)	21.98 ± 2.21	22.32 ± 2.23	22.44 ± 2.01	1.28	0.83 (NS)
Weight (kg)	64.08 ± 14.09	62.56 ± 11.87	64.44 ± 11.81	2.12	0.71 (NS)
Height (cm)	163.22 ± 9.68	160.66 ± 7.61	162.68 ± 8.47	1.78	0.81 (NS)
BMI (kg/m²)	22.91 ± 2.11	22.15 ± 2.74	22.28 ± 2.72	1.68	0.79 (NS
Waist circumference (cm)	85.21 ± 3.17	84.34 ± 3.21	84.19 ± 3.09	1.46	0.82 (NS)
Hip circumference (cm)	96.28 ± 2.45	97.12 ± 2.07	96.71 ± 2.11	2.09	0.70 (NS)
Waist-to-hip ratio	0.88 ± 0.11	0.86 ± 0.16	0.87 ± 0.12	2.11	0.71 (NS)

Statistical analysis

Data were analyzed using SPSS, Microsoft Excel, and GraphPad Prism 5 software. One-way ANOVA was used for intergroup comparisons. Where analysis of variance (ANOVA) showed statistical significance, Tukey’s post-hoc test was applied for multiple comparisons. In addition to *P*-values, effect size (eta-squared, *η*²) was calculated to quantify the magnitude of group differences. Student’s t-test was used where appropriate, following standard biostatistical methods [8].

All tests were two-tailed. A *P*-value < 0.05 was considered statistically significant, and *P* < 0.001 was considered highly significant.

Ethics statement

The study was approved by the Institutional Ethics Committee, Government Medical College, Nagpur (IEC approval number: 1152). Written informed consent was obtained from all participants before enrollment.

The study involved minimal-risk procedures. Participants were informed that spirometry testing may be associated with mild, transient discomfort, including coughing, lightheadedness, or fatigue. All testing was conducted under supervision, and participants were free to discontinue testing at any time if they experienced discomfort.

No financial compensation was provided for participation. Participation was entirely voluntary, and participants were informed that they could withdraw from the study at any time without any penalty or effect on their medical care or academic status.

## Results

Participant enrollment and data completeness

All 150 enrolled participants completed the study, and complete data were available for analysis.

Anthropometric characteristics

A total of 150 participants completed the study, with complete data available for analysis. There were no statistically significant differences in age, weight, height, body mass index, waist circumference, hip circumference, or waist-to-hip ratio among swimmers, runners, and sedentary controls, indicating comparability of the study groups with respect to baseline anthropometric characteristics (Table 1).

Pulmonary function parameters

The comparison of pulmonary function parameters among swimmers, runners, and sedentary controls demonstrated statistically significant intergroup differences for all measured variables (*P* < 0.0001). Forced vital capacity and forced expiratory volume in one second showed progressively higher mean values from sedentary controls to runners and swimmers. A similar trend was observed for the FEV₁/FVC ratio, indicating better ventilatory efficiency in physically active participants. Maximum voluntary ventilation and peak expiratory flow rate were also significantly higher in swimmers, followed by runners and sedentary controls, reflecting superior respiratory muscle performance in trained individuals (Table 2).

**Table 2:** Comparison of pulmonary function parameters among study groups. Values are expressed as mean ± standard deviation (SD). Intergroup comparison was performed using one-way analysis of variance (ANOVA). A *P*-value < 0.05 was considered statistically significant. *Statistically significant differences (*P* < 0.05). FVC, forced vital capacity; FEV₁, forced expiratory volume in one second; MVV, maximum voluntary ventilation; PEFR, peak expiratory flow rate

Parameter	Swimmers (*n *= 50)	Runners (*n *= 50)	Sedentary (*n *= 50)	*F*-value	*P*-value^*^
FVC (L)	3.07 ± 0.65	2.92 ± 0.63	2.64 ± 0.63	5.87	<0.0001
FEV_1_ (L)	2.84 ± 0.52	2.65 ± 0.61	2.30 ± 0.51	12.47	<0.0001
FEV_1_/FVC (%)	92.50 ± 5.53	90.75 ± 6.02	87.11 ± 5.29	11.96	<0.0001
MVV (L/minute)	119.02 ± 7.83	117.18 ± 6.89	114.38 ± 10.61	12.96	<0.0001
PEFR (L/second)	7.29 ± 1.05	7.26 ± 1.01	7.20 ± 1.07	17.18	<0.0001

## Discussion

This study compared pulmonary function parameters among recreational swimmers, runners, and sedentary healthy male adults to evaluate sport-specific respiratory adaptations in a non-elite population. The findings indicate that recreational swimmers demonstrated higher lung volumes and spirometric indices compared to sedentary individuals, while runners exhibited intermediate values. These observations support the concept that habitual physical activity influences pulmonary function, with variations depending on the nature of the activity performed [1,3,6].

Previous studies have consistently reported superior pulmonary function in swimmers when compared to sedentary controls. Akhade and Muniyappanavar documented significantly higher pulmonary function parameters in swimmers than in sedentary individuals, suggesting that swimming is associated with enhanced respiratory capacity [2]. Similarly, Vedala et al. observed better pulmonary function in physically active populations compared to sedentary subjects, reinforcing the beneficial effect of regular physical activity on respiratory parameters [1]. The findings of the present study are in agreement with these reports.

Comparative studies evaluating different forms of physical activity suggest that swimming may confer additional respiratory advantages compared to land-based exercises. Doherty and Dimitriou demonstrated larger lung volumes in swimmers compared to land-based athletes and sedentary controls using allometric scaling, indicating that these differences are not solely attributable to body size [6]. The higher pulmonary function values observed among recreational swimmers in the present study are consistent with these findings, despite differences in training intensity and competitive level.

The distinctive breathing pattern associated with swimming, involving controlled breathing, breath-holding, and breathing against water resistance, has been proposed as a mechanism contributing to improved pulmonary function. Enhanced respiratory muscle strength and ventilatory efficiency may result from these repeated respiratory demands. Segizbaeva and Aleksandrova reported greater respiratory muscle strength and improved ventilatory outcomes in trained individuals compared to untrained persons, supporting this physiological explanation [4].

Runners in the present study demonstrated better pulmonary function parameters than sedentary individuals, which aligns with findings from studies examining the effects of moderate aerobic exercise. Okon et al. reported improved pulmonary function among young adults participating in regular aerobic activity, indicating that endurance exercise positively influences respiratory function [3]. However, unlike swimming, running does not consistently produce marked increases in lung volumes, which may explain the intermediate values observed in runners.

Some variability in pulmonary function outcomes across studies may be attributed to differences in participant characteristics, training duration, exercise intensity, and assessment techniques. Lazović-Popović et al. highlighted that respiratory adaptations vary between athletes and non-athletes depending on the type of sport and training exposure [5]. These factors should be considered when interpreting inter-study differences and comparing results across populations.

It is also important to consider alternative explanations for the observed group differences. Individuals with inherently larger lung volumes or better baseline respiratory capacity may be more likely to self-select into swimming or endurance activities, which could contribute to the observed differences independent of training effects. In addition, because pulmonary function outcomes were not adjusted for anthropometric determinants or training intensity, and residual confounding cannot be excluded, the observed differences may reflect a combination of training-related adaptations and pre-existing physiological characteristics. Therefore, the findings should be interpreted as exploratory and hypothesis-generating rather than definitive evidence of causal training effects.

A key strength of the present study is its focus on recreational athletes, a group that is less frequently studied than elite performers. By including swimmers, runners, and sedentary individuals, the study provides a balanced comparison of commonly practiced physical activities. However, the cross-sectional design limits causal inference, and the possibility of self-selection and residual confounding must be considered. Longitudinal studies are needed to determine whether the observed differences are primarily a result of training or reflect pre-existing physiological characteristics.

Limitations

The present study did not account for sport-specific training variables such as training intensity, swimming stroke type, running pace, or environmental conditions during training. These factors are known to influence respiratory adaptations and may have contributed to inter-individual variability in pulmonary function parameters. In addition, due to the cross-sectional design, temporal and causal relationships between exercise modality and pulmonary function cannot be established, and the observed differences should be interpreted as associations rather than cause-and-effect relationships.

Furthermore, despite efforts to control for major confounders through eligibility criteria, important potential sources of residual confounding were not systematically assessed or controlled for, including environmental air pollution exposure, socioeconomic status, dietary patterns, sleep habits, and variability in actual training intensity and adherence. These factors are known to influence pulmonary function and may have contributed to variability in the results, limiting attribution of observed differences solely to exercise modality.

Pulmonary function is influenced by multiple interacting physiological, environmental, and behavioral factors, and therefore, complete control of all relevant determinants was not feasible in this multifactorial physiological study design.

Pulmonary function outcomes were not statistically adjusted for anthropometric determinants such as height, body mass index, or body surface area, which are strong predictors of lung volumes. In addition, formal statistical tests of normality and homogeneity of variance (e.g., Shapiro-Wilk and Levene’s tests) were not performed, and assumptions of parametric testing were based on descriptive assessment and visual inspection.

No formal adjustment for multiple testing (e.g., Bonferroni or related corrections) was applied, which may increase the risk of Type I error. Therefore, findings should be interpreted with appropriate caution, particularly given the exploratory nature of the analyses.

Although effect size was reported using eta-squared (η²), additional effect size measures such as Cohen’s d and confidence intervals were not calculated, which limits assessment of the clinical or practical significance of the findings.

The study population was restricted to healthy young adult males, which limits external validity and generalizability of the findings and does not account for potential sex- and age-related physiological differences in pulmonary function. As this study involved recreationally active participants, the primary objective was a comparison between activity groups rather than a detailed characterization of training load.

Future longitudinal studies incorporating standardized measures of training intensity and volume, formal testing of statistical assumptions, multivariable adjustment for anthropometric and environmental factors, correction for multiple comparisons, and inclusion of female participants and broader age ranges are warranted to better elucidate causal effects and dose-response relationship.

## Conclusions

This study demonstrates that recreational swimmers and runners exhibit significantly better pulmonary function parameters compared with sedentary healthy male adults. Regular engagement in aerobic physical activity is associated with improved lung volumes, airflow indices, and ventilatory efficiency, even in non-elite populations.

Recreational swimming appears to confer distinct respiratory benefits, likely due to controlled breathing patterns, breath-holding, and increased inspiratory effort against water resistance. Recreational running also results in favorable pulmonary adaptations through sustained ventilatory demand, rhythmic breathing, and enhanced respiratory muscle conditioning. In contrast, sedentary healthy male adults show comparatively lower pulmonary function values, which may be attributed to the absence of regular ventilatory stimulation and reduced respiratory muscle engagement.

These findings highlight that both aquatic and land-based aerobic activities are effective in promoting respiratory health in recreationally active individuals. However, given the cross-sectional design and restricted study population, future research should employ longitudinal study designs to better establish temporal and causal relationships. Future studies should also include female participants, broader age groups, and individuals with respiratory disease to improve generalizability. In addition, incorporation of objective measures of training intensity and volume, multivariable adjustment for key confounders, and more comprehensive pulmonary assessments (including respiratory muscle strength and diffusion capacity) would further strengthen understanding of exercise-related respiratory adaptations.

## References

[1] Vedala SR, Paul N, Mane AB (2013). Differences in pulmonary function tests among physically active and sedentary populations. Natl J Physiol Pharm Pharmacol.

[2] Akhade VV, Muniyappanavar N (2014). Comparative study of pulmonary functions in swimmers and sedentary controls. Natl J Physiol Pharm Pharmacol.

[3] Okon IA, Okorocha AE, Beshel JA, Abali HC, Owu DU (2023). Pulmonary functions and anthropometric parameters of young male and female adults participating in moderate aerobic exercise. Curr Res Physiol.

[4] Segizbaeva MO, Aleksandrova NP (2021). Respiratory muscle strength and ventilatory function outcome: Differences between trained athletes and healthy untrained persons. Adv Exp Med Biol.

[5] Lazovic-Popovic B, Zlatkovic-Svenda M, Durmic T, Djelic M, Djordjevic Saranovic S, Zugic V (2016). Superior lung capacity in swimmers: some questions, more answers!. Rev Port Pneumol (2006).

[6] Doherty M, Dimitriou L (1997). Comparison of lung volume in Greek swimmers, land based athletes, and sedentary controls using allometric scaling. Br J Sports Med.

[7] (1995). Standardization of spirometry, 1994 update. American Thoracic Society. Am J Respir Crit Care Med.

[8] Mahajan BK, Khanal AB (2016). Methods in Biostatistics for Medical Students and Research Workers, 8th edn. https://www.jaypeebrothers.com.

